# Subjective fatigue in individuals with anxiety and mood disorders correlates with specific traits of obsessive-compulsive personality disorder

**DOI:** 10.1016/j.nsa.2024.104048

**Published:** 2024-02-15

**Authors:** Agne Stanyte, Naomi A. Fineberg, Aurelija Podlipskyte, Julija Gecaite-Stonciene, Julius Burkauskas

**Affiliations:** a Laboratory of Behavioral Medicine, Neuroscience Institute, Lithuanian University of Health Sciences, Kaunas-Palanga, Lithuania; b University of Hertfordshire, Hatfield, UK; c National Obsessive Compulsive Disorders Specialist Service, Hertfordshire Partnership University, NHS Foundation Trust, Welwyn Garden City AL8 6HG, UK; d University of Cambridge Clinical Medical School, Cambridge, UK

**Keywords:** Obsessive compulsive personality, Anankastia, Fatigue, Anxiety, Depression, Common mental disorders

## Abstract

Fatigue is a common debilitating symptom in individuals with anxiety and mood disorders, also known as common mental disorders (CMD). Aspects of fatigue are seen to be independently associated with the presence of obsessive-compulsive personality disorder (OCPD) in individuals with CMD. However, the relationship between different traits of OCPD and fatigue is still under-researched. Therefore, the aim of our study was to examine the associations between individual OCPD traits and fatigue in those with CMD. This cross-sectional study investigated 203 individuals (76.8% female, mean age 40.8 ± 11.8) attending a stress-related disorders day care unit. Participants were evaluated for OCPD traits by using the Compulsive Personality Assessment Scale and completed the Multidimensional Fatigue Inventory-20. Out of 203 participants, 42 (20.7%) fulfilled operational criteria for OCPD (73.8% female). Participants with OCPD had greater reduced motivation, mental and physical fatigue scores than those without OCPD (13.2 ± 3.6 vs. 14.5 ± 3.6, p = 0.035; 14.4 ± 4.3 vs. 16.1 ± 3.5, p = 0.023; 13.5 ± 4.3 vs. 15.0 ± 4.3, p = 0.049, respectively). In individuals with OCPD, higher levels of rigidity correlated with physical fatigue (ρ = 0.463, p = 0.002) and reduced activity (ρ = 0.363, p = 0.018), while preoccupation with details was associated with reduced motivation (ρ = 0.437, p = 0.004). In conclusion, in individuals with CMD and comorbid OCPD, several OCPD traits were significantly related to subjective measures of fatigue. As traits are considered stable features, there results suggest they might play a causative role in generating subjective fatigue in CMD.

## 1 Introduction

Obsessive-compulsive personality disorder (OCPD) is a personality disorder described as an inflexible and maladaptive pattern of preoccupation with orderliness, perfectionism and need for control (American Psychiatric Association, 2013). Individuals with OCPD can be characterised as having executive dysfunction, cognitive inflexibility (Fineberg et al., 2015; Marincowitz et al., 2022; Paast et al., 2016), high levels of interpersonal and psychological distress (Paast et al., 2016; Solomonov et al., 2020). These symptoms tend to remain stable over time and cause functional and social impairments in everyday life (Skodol et al., 2005). Importantly, OCPD is often complicated by the secondary development of anxiety and mood disorders, also known as common mental disorders (CMD). OCPD is considered to be one of the most prevalent personality disorders in the general population (Winsper et al., 2020; Volkert et al., 2018; Burkauskas et al., 2020), with the prevalence being even higher in clinical and psychiatric samples (Clemente et al., 2022), especially in individuals with CMD (Kovanicova et al., 2020).

Fatigue is a core symptom of CMD (Chung et al., 2015; Ferentinos et al., 2009; Leung et al., 2022; Mozuraityte et al., 2023), usually reflecting subjective feelings of exhaustion, decreased energy and capacity for physical or mental work (Ghanean et al., 2018; Smets et al., 1995). In individuals with CMD, fatigue predicts functional limitations, reduced quality of life (Corfield et al., 2016), and lower probability of achieving remission in depression (Robinson et al., 2015; Stutz et al., 2019). Fatigue in CMD is difficult to treat, as it does not respond to effective antidepressant treatment (Chung et al., 2015) and becomes the main residual symptom even after reaching remission (Chung et al., 2015; Targum et al., 2011).

Since CMD and OCPD are closely related, it can be hypothesised that OCPD also plays a causative role in fatigue. There have been few studies that link fatigue with the traits that are related to OCPD in more broad and general populations. For example, workaholism, usually defined as maladaptive, compulsive feelings, thoughts and behaviours about work, has been associated with fatigue in a sample of employees and their spouses (Clark et al., 2021). This type of relationship tends to lead towards reduced cognitive flexibility, meaning that people under fatigue tend to use more automatic regulatory processes to guide their actions and ideas, which likely results in rigid work behaviours (Clark et al., 2021; van Wijhe et al., 2014), which is also a characteristic seen in individuals with OCPD (Fineberg et al., 2015; Atroszko et al., 2020). Workaholism is also predicted to mediate the relationship between burnout and perfectionism between workers (Falco et al., 2014; Taris et al., 2010), workers with perfectionism being frequently linked with physical and mental fatigue in student populations (Dittner et al., 2011), and individuals with multiple sclerosis (Besharat et al., 2011). Furthermore, perfectionism is associated with increased depressive symptoms in individuals with chronic fatigue syndrome (Wright et al., 2021).

Whereas fatigue has been previously associated with various constituent traits of OCPD, the relationship between specific OCPD traits and fatigue has not been systematically studied. A study by Gecaite-Stonciene et al. (2020) examining the presence of OCPD in individuals with CMD found that the presence of OCPD was significantly associated with mental fatigue, but not other fatigue characteristics. Given the certain similarities between OCPD symptoms and fatigue, namely behavioural inhibition and executive planning (Fineberg et al., 2015; Fava, 2003; Plukaard et al., 2015), there is a need to clarify the relationship between OCPD traits and fatigue to identify fatigue-specific traits and better understand the mechanisms leading to more negative outcomes. Therefore, the aim of our study is to examine the associations between OCPD traits and fatigue in individuals with CMD.

## 2 Methods

### 2.1 Study participants

A cross-sectional study design was used to conduct the study with participants who were attending an outpatient stress-related disorders unit of the Neuroscience Institute Palanga Hospital at the Lithuanian University of Health Sciences, from April 2018 to September 2022. In order to participate in the study, the individuals had to be older than 18 years old and have a current diagnosis of anxiety and/or mood disorder, as established according to the *Diagnostic and Statistical Manual of Mental Disorders*, 5th edition (DSM-5) diagnostic criteria (American Psychiatric Association, 2013), using the Mini-International Neuropsychiatric Interview (M.I.N.I. 7.0.2) (Sheehan et al., 1998). Individuals who had current severe somatic illness (e.g., cancer), psychotic symptoms, cognitive impairment, high suicidal risk or did not speak fluent Lithuanian were excluded from the study. All participants received standard treatment for their diagnosis according to their clinical needs, including psychopharmacological treatment, psychological counselling, group psychotherapy and other psychotherapeutic interventions.

Out of 231 eligible participants, 28 (12.1%) did not have a current diagnosis of CMD, therefore were not included in the study.

### 2.2 Study procedure and measures

The Lithuanian Biomedical Research Ethics Committee approved the study protocol and procedure (reference No. B-2-38). The study was conducted in accordance with the Declaration of Helsinki. Before being included in the study, each participant signed an informed consent form.

Sociodemographic and clinical information, including diagnosis (as defined by M.I.N.I. 7.0.2), age, sex, education, history of smoking, and current medication use were collected during the first five days of admission to the unit.

The presence and severity of OCPD traits were measured using the Compulsive Personality Assessment Scale (CPAS) (Fineberg et al., 2007). CPAS is an observer-rated scale based on a semi-structured interview. CPAS has eight questions, each of which are based on the DSM-5 diagnostic criteria. Each question is scored on a scale of zero to four and the maximum score of the CPAS is 32. Consistent with the DSM-5 (American Psychiatric Association, 2013), the diagnosis of OCPD in our research was defined as a score of three (severe) or four (very severe) on at least four of the CPAS items. Cronbach's alpha for CPAS was 0.739.

The severity of subjective fatigue characteristics was measured using the Multidimensional Fatigue Inventory-20 (MFI-20) (Smets et al., 1995). The MFI-20 is a 20-item self-report measurement of fatigue. It includes five dimensions of general fatigue, physical fatigue, mental fatigue, reduced activity and reduced motivation. Each domain consists of four items which are rated on a 5-point Likert scale ranging from 1 (“yes, that is true”) to 5 (“no, that is not true”). The domain of general fatigue is composed of the general statements about fatigue and reduced functioning, covering physical as well as psychological aspects of fatigue (Cronbach's alpha 0.807). Physical fatigue concerns physical feelings related to fatigue (Cronbach's alpha 0.837), while mental fatigue refers to cognitive functioning, such as concentration difficulties (Cronbach's alpha 0.848). The reduced activity dimension evaluates the impact of psychological and physical factors on the activity level (Cronbach's alpha 0.811) and the reduced motivation domain assesses the lack of motivation to start an activity (Cronbach's alpha 0.639). The total score ranges from 4 to 20 on each sub-scale and 20 to 100 for total fatigue score. A higher overall score indicates higher levels of fatigue.

Objective fatigue characteristics were evaluated using an exercise capacity workload (EC) test. EC was measured using a standardised computer-driven bicycle ergometer with rising workload by 25 W (W) every 3 min (Fletcher et al., 2013). The peak of workload (PW) in watts (W) at the time of the end of the exercise test was considered to reflect EC.

### 2.3 Statistical analyses

Statistical analyses were conducted using the IBM SPSS Statistics for Windows (version 27; SPSS Inc, Chicago, IL, USA). Descriptive statistics were used to explore the samples' clinical and sociodemographic characteristics. Two-tailed Student's t-test for continuous variables and the χ^2^ test or Fisher's Exact test for categorical variables were used to compare sociodemographic, clinical and fatigue characteristics in study participants with and without OCPD. Spearman correlations were used to test associations between OCPD trait and fatigue characteristics. Here, the Benjamini-Hochberg correction was applied to control the false discovery rate, setting the p-value at 0.02.

## 3 Results

A total of 203 individuals were included in the study (77% female) with a mean age of 40.8 ± 11.8 years. Half of the study participants had a comorbid anxiety and mood disorder diagnosis (53.2%), 23.6% of individuals had a mood disorder diagnosis, and 23.2% - anxiety disorder diagnosis. Detailed characteristics of study participants are presented in Table 1.

**Table 1:** Baseline characteristics and cross sectional clinical and cognitive measurements for study participants, comparing the sub-groups with and without obsessive-compulsive personality disorder (OCPD)^a^.

	Total (N = 203)	Group without OCPD (N = 161)	Group with OCPD* (N = 42)	p-value
**Age, mean ± SD**	40.8 ± 11.8	40.4 ± 12.1	42.2 ± 11.0	0.395
**Gender, n (%)**				0.600
Men	47 (23.2)	36 (22.4)	11 (26.2)	
Women	156 (76.8)	125 (77.6)	31 (73.8)	
**Education, n (%)**				0.882
Tertiary education	55 (27.1)	44 (27.3)	11 (26.2)	
College/university degree	148 (72.9)	117 (72.7)	31 (73.8)	
**Diagnosis, n (%)**				
Major depressive disorder	42 (20.7)	31 (19.3)	11 (26.2)	0.392
Bipolar disorder	6 (3.0)	6 (3.7)	0 (0.0)	0.384
Major depressive disorder and anxiety disorder^b^	108 (53.2)	81 (50.3)	27 (64.3)	0.170
Generalized anxiety disorder	20 (9.9)	18 (11.2)	2 (4.8)	0.375
Panic disorder, agoraphobia	17 (8.4)	16 (9.9)	1 (2.4)	0.466
Social anxiety disorder	2 (1.0)	2 (1.2)	0 (0.0)	1.00
**History of smoking, n (%)**	56 (27.6)	48 (29.8)	8 (19.0)	0.164
**Fatigue as measured with MFI-20, mean ± SD**				
General fatigue	15.2 ± 3.9	14.9 ± 4.0	16.1 ± 3.3	0.071
Physical fatigue	13.8 ± 4.3	13.5 ± 4.3	15.0 ± 4.3	0.049
Reduced activity	14.4 ± 4.2	14.1 ± 4.1	15.5 ± 4.3	0.069
Reduced motivation	13.4 ± 3.6	13.2 ± 3.6	14.5 ± 3.6	0.035
Mental fatigue	14.8 ± 4.2	14.4 ± 4.3	16.1 ± 3.5	0.023

OCPD – Obsessive-compulsive personality disorder; MFI-20 – Multidimensional Fatigue Inventory-20.

Note.

a Defined operationally using the Compulsive Personality Assessment Scale.

b Generalized anxiety disorder or panic disorder or agoraphobia or social anxiety disorder.

Out of the 203 participants, 42 (20.7%) fulfilled operational criteria for OCPD, defined by using the clinician-administered CPAS (Fineberg et al., 2007). Participants with OCPD were compared to participants without OCPD regarding demographic, clinical and subjective fatigue characteristics. The groups did not differ from one another regarding age, gender, diagnosis, and education (all p-values ≥0.05). The groups also did not differ from one another regarding current medication use (see Supplemental Table S1). Comparing fatigue characteristics, our results show that participants with OCPD had significantly higher levels of physical (p = 0.049), mental fatigue (p = 0.023) and reduced motivation (p = 0.035) compared to those without OCPD.

Next, we analysed the correlations between OCPD traits and fatigue in individuals with OCPD (Table 2). In individuals with OCPD, higher levels of rigidity correlated with physical fatigue (ρ = 0.463, p = 0.002) and reduced activity (ρ = 0.363, p = 0.018), while preoccupation with details correlated with reduced motivation (ρ = 0.437, p = 0.004).

**Table 2:** Correlations between Obsessive-compulsive personality disorder traits and fatigue.

	General fatigue	Physical fatigue	Reduced activity	Reduced motivation	Mental fatigue	Exercise capacity
Preoccupation with details	0.177 (0.263)	0.204 (0.194)	0.254 (0.105)	0.437 (0.004)*	0.170 (0.282)	0.122 (0.520)
Perfectionism	0.056 (0.725)	−0.096 (0.545)	0.105 (0.508)	0.145 (0.359)	0.161 (0.307)	0.137 (0.470)
Workaholism	0.216 (0.170)	0.223 (0.156)	0.219 (0.163)	0.056 (0.725)	*0.353 (0.022)*	0.138 (0.206)
Over-conscientiousness	−0.020 (0.901)	0.213 (0.176)	−0.034 (0.829)	0.008 (0.958)	0.214 (0.174)	−0.027 (0.885)
Hoarding	0.074 (0.639)	−0.047 (0.769)	−0.010 (0.947)	−0116 (0.465)	−0.171 (0.279)	0.181 (0.340)
Control	0.031 (0.846)	−0.055 (0.731)	0.151 (0.341)	0.078 (0.624)	0.063 (0.693)	0.107 (0.572)
Miserliness	−0.108 (0.495)	−0.122 (0.440)	−0.201 (0.203)	−0.187 (0.237)	−0.165 (0.297)	0.285 (0.127)
Rigidity	0.297 (0.056*)*	0.463 (0.002)*	0.363 (0.018)*	0.176 (0.266)	0.189 (0.232)	−0.268 (0.152)

Note: Statistically significant differences in bold based on Benjamini-Hochberg correction.

## 4 Discussion

The results of our cross-sectional study revealed that OCPD traits were significantly related with measures of subjective fatigue in individuals with CMD and comorbid OCPD. Our results highlight the impact of OCPD on subjective fatigue in a specific CMD population.

Our findings show that OCPD trait rigidity was associated with a higher prevalence of subjective physical fatigue and reduced activity. To the best of our knowledge, this is a novel finding that has not been reported before in related literature. It could be hypothesised that the associations are present due to the behavioural rigidity of individuals with OCPD. Rigid people have problems adapting to change, which can lead to behavioural inefficiency and higher physical demands in comparison to those who flexibly adapt behaviour according to current contingencies. At a cognitive level, fatigue often results in rigidity, characterised by difficulties flexibly switching between behavioural tasks, using more automatic cognitive processes, and overall cognitive inflexibility (Plukaard et al., 2015; van der Linden et al., 2003). In addition, for individuals with OCPD, difficulties in executive functions including cognitive flexibility (Fineberg et al., 2015) can become even more pronounced when experiencing subjective feelings of fatigue (Abd-Elfattah et al., 2015).

Secondly, we found that preoccupation with details, reflecting poor central coherence, was associated with reduced motivation to start new activities (Smets et al., 1995). This finding is also novel. A possible explanation of such relationship can be found in the results of a study by Fineberg et al. (2022). The mentioned study showed that preoccupation with details was associated with more adjustment problems to the easing of the COVID-19 restrictions in a general adult population (Fineberg et al., 2022). A possible explanation of such a relationship is that when individuals become excessively preoccupied with details, they may become overwhelmed by the perceived magnitude of the task at hand. The focus on every small aspect can make the task seem more daunting and unmanageable. As a result, they may feel a sense of overwhelm and avoidance, leading to reduced motivation to engage in the task. This can especially surface in the context of depression when the overall energy levels are reduced.

However, the differences in fatigue between the groups were rather small, preventing us from making straight forward clinical interpretations of study findings. Nevertheless, correlations between OCPD and fatigue were detected in only some of the traits, suggesting that these results reflect a unique relationship between OCPD and subjective fatigue in individuals with CMD. In the current sample of individuals with CMD and comorbid OCPD, objective fatigue, as measured by EC, was not significantly associated with OCPD traits, meaning that the underlying mechanisms could be related to psychological interactions between study variables. Research suggests that psychological treatments, such as Cognitive Behavioural Therapy (CBT), help reduce depression and anxiety symptoms in individuals with OCPD (Diedrich et al., 2015). In a specific sample of individuals with CMD and comorbid OCPD, CBT techniques could also be valuable to target the symptom of fatigue as well, including psychoeducation about fatigue and behavioural activation in order to diminish the symptoms of fatigue and enhance the motivation to continue treatment. However, in order to make any firm conclusions, future research should explore the possible mechanisms underlying the relationship between OCPD and fatigue further.

The findings of this study have to be interpreted in the light of their limitations. First, our selected cross-sectional study design prevents us from making implications with regards to causal effects between study variables. Second, the study was completed in a convenience sample of individuals from a single stress-related disorders unit, making it harder to generalise results to a broader population of individuals with CMD. Third, correlations between OCPD traits and fatigue were not controlled for the impact of CMD, the severity of the disorders or the use of medications. Last, the Benjamini-Hochberg criterion was used to control the false discovery rate. Even though this criterion is considered to be a liberal adjustment method for correcting for multiple comparisons, it is recommended as an alternative to Bonferroni-type adjustments in health studies (Glickman et al., 2014). These limitations should be addressed in future research, for example, recruiting more individuals with CMD and comorbid OCPD could help to confirm the findings and overcome these limitations.

## 5 Conclusions

Overall, our study demonstrates that in individuals with CMD and comorbid OCPD, certain OCPD traits were significantly related to subjective measures of fatigue. As traits are considered to be stable features, these results suggest they play a causative role in generating subjective fatigue in individuals with CMD. However, future studies should look at the brain-based mechanisms by which these variables may interact.

## Declaration of competing interest

The authors declare the following financial interests/personal relationships which may be considered as potential competing interests:1) Julija Gecaite-Stonciene works as a consultant at FACITtrans. 2) Julius Burkauskas served as a consultant at IQVIA. 3) Naomi A. Fineberg reports in the past 5 years personal fees from Taylor and Francis, Oxford University Press, Global Mental Health Academy and Elsevier; personal fees and non-financial support from Sun; non-financial support from RC PSYCH, CINP, WPA, the International Forum of Mood and Anxiety Disorders, ECNP and the Indian Association of Biological Psychiatry; grants from the Wellcome, UKRI, Orchard and NIHR; grants and non-financial support from EU COST Action; payment for consultancy from the UK MHRA, all outside of the submitted work.

## References

[bib1] Abd-Elfattah H.M., Abdelazeim F.H., Elshennawy S. (2015). Physical and cognitive consequences of fatigue: a review. J. Adv. Res..

[bib2] American Psychiatric Association (2013).

[bib3] Atroszko P.A., Demetrovics Z., Griffiths M.D. (2020). Work addiction, obsessive-compulsive personality disorder, burn-out, and global burden of disease: implications from the ICD-11. Int. J. Environ. Res. Publ. Health.

[bib4] Besharat M.A. (2011). Perfectionism and fatigue in multiple sclerosis. Psychol. Health.

[bib5] Burkauskas J., Fineberg N.A., Grant Jon E., Pinto Anthony, Chamberlain Samuel R. (2020). Obsessive-compulsive Personality Disorder/.

[bib6] Chung K.F., Yu Y.M., Yeung W.F. (2015). Correlates of residual fatigue in patients with major depressive disorder: the role of psychotropic medication. J. Affect. Disord..

[bib7] Clark M.A., Hunter E.M., Carlson D.S. (2021). Hidden costs of anticipated workload for individuals and partners: exploring the role of daily fluctuations in workaholism. J. Occup. Health Psychol..

[bib8] Clemente M.J. (2022). A meta-analysis and meta-regression analysis of the global prevalence of obsessive-compulsive personality disorder. Heliyon.

[bib9] Corfield E.C., Martin N.G., Nyholt D.R. (2016). Co-occurrence and symptomatology of fatigue and depression. Compr. Psychiatr..

[bib10] Diedrich A., Voderholzer U. (2015). Obsessive-compulsive personality disorder: a current review. Curr. Psychiatr. Rep..

[bib11] Dittner A.J., Rimes K., Thorpe S. (2011). Negative perfectionism increases the risk of fatigue following a period of stress. Psychol. Health.

[bib12] Falco A. (2014). "The best or nothing": the mediating role of workaholism in the relationship between perfectionism and burnout. TPM - Test. Psychometrics, Methodol. Appl. Psychol..

[bib13] Fava M. (2003). Symptoms of fatigue and cognitive/executive dysfunction in major depressive disorder before and after antidepressant treatment. J. Clin. Psychiatry.

[bib14] Ferentinos P.P. (2009). Fatigue and somatic anxiety in patients with major depression. Psychiatriki.

[bib15] Fineberg N.A. (2007). Does obsessive-compulsive personality disorder belong within the obsessive-compulsive spectrum?. CNS Spectr..

[bib16] Fineberg N.A. (2015). The neuropsychology of obsessive-compulsive personality disorder: a new analysis. CNS Spectr..

[bib17] Fineberg N.A. (2022). Individual obsessive-compulsive traits are associated with poorer adjustment to the easing of COVID-19 restrictions. J. Psychiatr. Res..

[bib18] Fletcher G.F. (2013). Exercise standards for testing and training: a scientific statement from the American Heart Association. Circulation.

[bib19] Gecaite-Stonciene J. (2020). Mental fatigue, but not other fatigue characteristics, as a candidate feature of obsessive compulsive personality disorder in patients with anxiety and mood disorders-an exploratory study. Int. J. Environ. Res. Publ. Health.

[bib20] Ghanean H., Ceniti A.K., Kennedy S.H. (2018). Fatigue in patients with major depressive disorder: prevalence, burden and pharmacological approaches to management. CNS Drugs.

[bib21] Glickman M.E., Rao S.R., Schultz M.R. (2014). False discovery rate control is a recommended alternative to Bonferroni-type adjustments in health studies. J. Clin. Epidemiol..

[bib22] Kovanicova M., Kubasovska Z., Pallayova M. (2020). Exploring the presence of personality disorders in a sample of psychiatric inpatients. Journal of Psychiatry and Psychiatric Disorders.

[bib23] Leung P., Li S.H., Graham B.M. (2022). The relationship between repetitive negative thinking, sleep disturbance, and subjective fatigue in women with Generalized Anxiety Disorder. Br. J. Clin. Psychol..

[bib24] Marincowitz C., Lochner C., Stein D.J. (2022). The neurobiology of obsessive-compulsive personality disorder: a systematic review. CNS Spectr..

[bib25] Mozuraityte K. (2023). Mental fatigue in individuals with psychiatric disorders: a scoping review. Int. J. Psychiatr. Clin. Pract..

[bib26] Paast N. (2016). Comparison of cognitive flexibility and planning ability in patients with obsessive compulsive disorder, patients with obsessive compulsive personality disorder, and healthy controls. Shanghai Arch Psychiatry.

[bib27] Plukaard S. (2015). Cognitive flexibility in healthy students is affected by fatigue: an experimental study. Learn. Indiv Differ.

[bib28] Robinson R.L. (2015). The importance of unresolved fatigue in depression: costs and comorbidities. Psychosomatics.

[bib29] Sheehan D.V. (1998). The Mini-International Neuropsychiatric Interview (M.I.N.I.): the development and validation of a structured diagnostic psychiatric interview for DSM-IV and ICD-10. J. Clin. Psychiatry.

[bib30] Skodol A.E. (2005). Stability of functional impairment in patients with schizotypal, borderline, avoidant, or obsessive-compulsive personality disorder over two years. Psychol. Med..

[bib31] Smets E.M. (1995). The Multidimensional Fatigue Inventory (MFI) psychometric qualities of an instrument to assess fatigue. J. Psychosom. Res..

[bib32] Solomonov N. (2020). Comparing the interpersonal profiles of obsessive-compulsive personality disorder and avoidant personality disorder: are there homogeneous profiles or interpersonal subtypes?. Personal Disord.

[bib33] Stutz P.V., Golani L.K., Witkin J.M. (2019). Animal models of fatigue in major depressive disorder. Physiol. Behav..

[bib34] Targum S.D., Fava M. (2011). Fatigue as a residual symptom of depression. Innov Clin Neurosci.

[bib35] Taris T.W., Van Beek I., Schaufeli W.B. (2010). Why do perfectionists have a higher burnout risk than other? The mediational effect of workaholism. Romanian Journal of Applied Psychology.

[bib36] van der Linden D., Frese M., Meijman T.F. (2003). Mental fatigue and the control of cognitive processes: effects on perseveration and planning. Acta Psychol..

[bib37] van Wijhe C.I., Peeters M.C.W., Schaufeli W.B. (2014). Enough is enough: cognitive antecedents of workaholism and its aftermath. Hum. Resour. Manag..

[bib38] Volkert J., Gablonski T.C., Rabung S. (2018). Prevalence of personality disorders in the general adult population in Western countries: systematic review and meta-analysis. Br. J. Psychiatry.

[bib39] Winsper C. (2020). The prevalence of personality disorders in the community: a global systematic review and meta-analysis. Br. J. Psychiatry.

[bib40] Wright A. (2021). Perfectionism, depression and anxiety in chronic fatigue syndrome: a systematic review. J. Psychosom. Res..

