# Supplementary material for: Subjective fatigue in individuals with anxiety and mood disorders correlates with specific traits of obsessive-compulsive personality disorder
Source: Neurosci Appl. 2024 Feb 15;3:104048. doi: 10.1016/j.nsa.2024.104048 (PMC12244212; doi:10.1016/j.nsa.2024.104048)
Supplement: Multimedia component 1 [file mmc1.docx]

Supplement

**Table S1**. medication use according to NbN approved drug class, comparing the sub-groups with and without obsessive compulsive personality disorder (OCPD)*

|  | **Total**  **(N=203)** | **Group without OCPD (N=161)** | **Group with OCPD***  **(N=42)** | **p-value** |
| --- | --- | --- | --- | --- |
| **Drugs for depression** | | | | |
| Melatonin, serotonin agonist-antagonist | 8 (5.6) | 6 (5.4) | 2 (6.7) | 0.863 |
| Serotonin | 104 (73.2) | 86 (76.8) | 18 (60.0) | 0.123 |
| Glutamate, opioid | 1 (0.7) | 0 (0.0) | 1 (3.3) | 0.544 |
| Norepinephrine, serotonin multimodal | 19 (13.4) | 14 (12.5) | 5 (16.7) | 0.774 |
| Serotonin, norepinephrine reuptake inhibitor | 5 (3.5) | 2 (1.8) | 3 (10.0) | 0.167 |
| Dopamine, norepinephrine reuptake inhibitor | 2 (1.4) | 2 (1.8) | 0 (0.0) | 0.896 |
| Serotonin multimodal | 3 (2.1) | 2 (1.8) | 1 (3.3) | 0.842 |
|  |  |  |  |  |
| **Drugs for anxiety** | | | | |
| GABA (Gamma-aminobutyric acid) | 65 (100) | 48 (100) | 17 (100) | 1.00 |
|  |  |  |  |  |
| **Drugs for relapse prevention** | | | | |
| Glutamate | 3 (37.5) | 0 (0.0) | 3 (60.0) | 0.356 |
| Glutamate channel blocker | 5 (62.5) | 3 (100) | 2 (40.0) | 0.356 |
|  |  |  |  |  |
| **Drugs for psychosis** | | | | |
| Dopamine receptor antagonist | 15 (27.8) | 14 (30.4) | 1 (12.5) | 0.518 |
| Dopamine, serotonin, norepinephrine multimodal | 37 (68.5) | 30 (65.2) | 7 (87.5) | 0.375 |
| Dopamine, serotonin receptor antagonist | 2 (3.7) | 2 (4.3) | 0 (0.0) | 0.712 |

Note: * defined operationally using the Compulsive Personality Assessment Scale

NbN- Neuroscience-based nomenclature; OCPD – Obsessive-compulsive personality disorder
